# Survey data on challenges and resilience capacities of smallholder farmers in Sri Lanka

**DOI:** 10.1016/j.dib.2026.113098

**Published:** 2026-07-24

**Authors:** Arani K. Ranasinghe, Lai Ming Lam, Kelly K. Miller, Brett A. Bryan

**Affiliations:** School of Life and Environmental Sciences, Deakin University, 221 Burwood Highway, Burwood, 3125, Victoria, Australia

**Keywords:** Agricultural challenges, Resilience, Small-scale farmers, Sri Lanka

## Abstract

This dataset contains raw data collected through household surveys of smallholder farmers on socioeconomic and farm characteristics, agricultural challenges they face, resilience capacities, resilience perceptions, and risk management strategies in cascade farming systems in the dry zone of Sri Lanka. Data were collected through face-to-face surveys with 300 smallholder farmers from 12 villages in the Galgamuwa Divisional Secretariat, Kurunegala District, Sri Lanka, using a pre-tested structured questionnaire. A simple random sampling approach was employed to select respondents. Farmers were randomly selected from each village using official voters’ lists of the respective village administrative divisions, and surveys were conducted between June and September 2025. Ethics approval for the survey was obtained from the Deakin University Human Research Ethics Committee. This dataset provides valuable insights into the complex challenges faced by smallholder farmers in dry-zone cascade systems in Sri Lanka, their resilience capacities and resilience perceptions, and their adaptive responses within dynamic social-ecological contexts. The dataset serves as a foundational reference for future longitudinal studies on resilience, enabling the assessment of how resilience-enhancing policies and adaptation interventions perform over time. The data are provided with this article.

Specifications Table

**Table:** 

Subject	Earth & Environmental Sciences
Specific subject area	Agricultural challenges and resilience of smallholder farming systems, focusing on farmers’ capacity to cope, adapt, and transform under stress and shocks
Type of data	Raw (categorical and numerical) data and analysed data (presented in tables, charts and figures). Dataset in Microsoft Excel (.xlsx) file format. The Excel file consists of six worksheets. The first worksheet contains the codebook. The second includes socio-economic and farm characteristics (31 variables), the third presents agricultural challenges (20 variables), the fourth contains resilience capacities (6 variables), the fifth includes resilience-related perceptions (24 variables), and the sixth presents risk management strategies (22 variables). All worksheets contain data from the same 300 respondents.
Data collection	Data were collected through face-to-face surveys with 300 farmers from twelve villages within the Galgamuwa Divisional Secretariat, using a structured questionnaire. Farmers were randomly selected from each village using official voters’ lists. The questionnaire was developed based on a literature review and the researchers’ contextual knowledge. Ethics approval was obtained from the Deakin University Human Research Ethics Committee. Before surveys, potential respondents were informed about the objectives and research procedures, and written consent was obtained. Only farmers were interviewed, as the study aimed to capture farmers' experiences, perceptions, knowledge, and challenges.
Data source location	Galgamuwa Divisional Secretariat, Kurunegala District, North-Western Province, Sri Lanka (7°59′11.4″N, 80°17′16.4″E).
Data accessibility	Repository name: Mendeley Data Data identification number: 10.17632/rynj38rnsd.2 Direct URL to data: https://data.mendeley.com/datasets/rynj38rnsd/2
Related research article	None

## 1 Value of the Data

• The dataset provides an empirical resource for future research on smallholder farming systems, supporting interdisciplinary research on agricultural resilience and contributing to a better understanding of farmer decision-making and resilience under multiple agricultural stressors.
• The data are valuable to policymakers, sustainable development practitioners, and government and non-governmental organisations, as they offer detailed, context-specific evidence to support the design of targeted interventions, strengthen agricultural policy, and inform programs aimed at enhancing resilience and adaptation in smallholder agriculture at local and regional levels.
• The dataset provides a baseline for future longitudinal resilience research, making it useful for evaluating the effectiveness of resilience-building policies and adaptation programs over time.
• The dataset is relevant for broader development and planning purposes, including local and regional land-use planning, community development initiatives, livelihood support programs, and interdisciplinary research on rural sustainability, given the central role of smallholder agriculture in the study area and across rural Sri Lanka.

## 2 Background

The data collection is grounded in the resilience framework proposed by Meuwissen et al. [1], which conceptualizes resilience of farming systems in terms of robustness, adaptability, and transformability, and considers environmental, economic, social, and institutional dimensions. Data were collected through structured, face-to-face surveys with 300 smallholder farmers, capturing information on perceived agricultural challenges, resilience capacities, resilience perceptions and risk management strategies using Likert-scale measurements. The survey instrument was designed to capture information on perceived agricultural challenges, resilience capacities, resilience perceptions, and risk management strategies. Responses were recorded using Likert-scale measurements to ensure consistency in capturing subjective perceptions. This dataset offers a systematic empirical foundation for analysing resilience-related aspects in smallholder agricultural systems.

## 3 Data Description

The dataset contains the responses of 300 smallholder mixed farmers in Galgamuwa Divisional Secretariat, North-Western Province in Sri Lanka, collected through face-to-face interviews. This dataset contains (1.1) socio-economic and demographic characteristics of the farmers, (1.2) smallholder farm characteristics, (1.3) agricultural challenges faced by farmers, (1.4) farmers’ resilience capacities, (1.5) farmers’ resilience perceptions, and (1.6) their current risk management strategies.

### 3.1 Socio-economic and demographic data

**Fig. 1:**
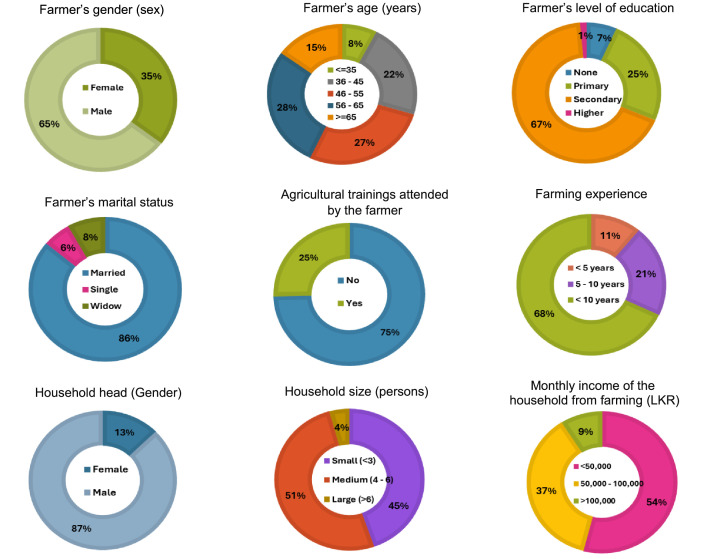
Socio-demographic characteristics of survey respondents.

The socio-economic characteristics of the respondents and their households were obtained using a series of multiple-choice questions. These variables included gender (sex), age, level of education, training received, and household income (Fig. 1). The complete dataset is provided as a supplementary file.

### 3.2 Small-holder farm characteristics

Information on several attributes describing the study participants, such as farm size, crops grown, income, and access to input and output markets, was collected using multiple-choice questions and is summarised in Table 1. Data are provided as a supplementary file.

**Table 1:** Smallholder farmer characteristics (*n* = 300).

Characteristics	Category	Frequency	Percentage (%)
Farm size	<1 acre	129	43.0
	1 – 2 acres	120	40.0
	Above 2 acres	51	17.0
Ownership of the land	Own land	240	80.0
	Leasehold land	42	14.0
	Common land	18	6.0
Household growing crops	Paddy/rice	271	90.3
	Vegetables	121	40.3
	Maize	36	12.0
	Mung-beans	50	16.6
	Groundnuts	27	9.0
	Fruits	51	17.0
	Others	46	15.3
Cash generation from livestock	Yes	59	19.6
	No	241	80.3
Types of animal rearing	Intensive	13	22.0
	Semi-extensive	29	49.2
	Extensive	17	28.8
Main water source for agriculture	Lake/reservoir	261	87.0
	Rainwater	44	14.6
	Agro-well	120	40.0
Using hired labor	Yes	216	72.0
	No	84	28.0
Having own vehicle to support agricultural activities	Yes	109	36.3
	No	191	63.6
Having easy access to input market	Yes	76	25.3
	No	224	74.6
Having easy access to output market	Yes	110	36.6
	No	190	63.3
Farm Type	Conventional	288	96.0
	Organic (not certified)	12	4.0
Having off-farm income	Yes	174	58.0
	No	126	42.0
Investment of off-farm income in agriculture	Yes	152	50.6
	No	148	49.3
Is main income agriculture	Yes	216	72.0
	No	84	28.0

### 3.3 Agricultural challenges faced by smallholder farmers

**Fig. 2:**
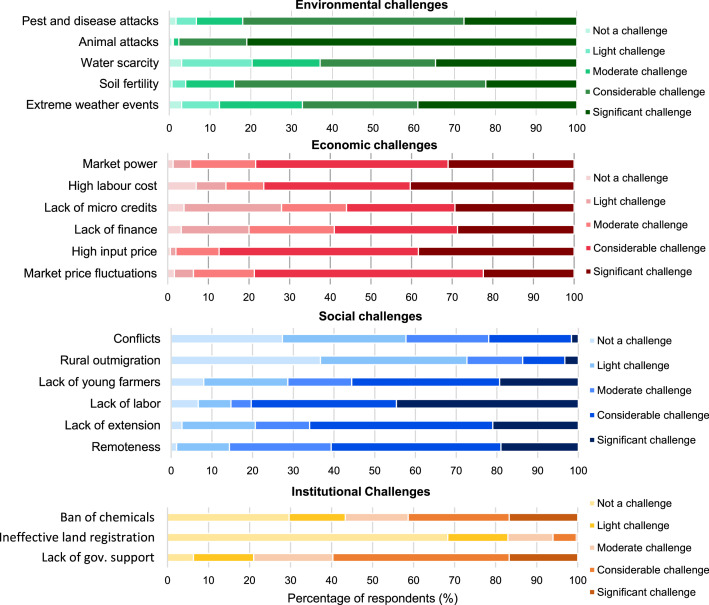
Farmers’ response to agricultural challenges.

A list of potential environmental, economic, social and institutional challenges (including both short-term shocks and long-term challenges) was developed based on a review of relevant literature and the researchers' contextual knowledge. During the survey, farmers were asked to indicate which challenges they face. Their responses, measured using a five-point Likert scale (1: Not a challenge, 2: Light challenge, 3: Moderate challenge, 4: Considerable challenge, 5: Significant challenge), are illustrated in Fig. 2.

### 3.4 Perceived resilience capacities of farmers

**Fig. 3:**
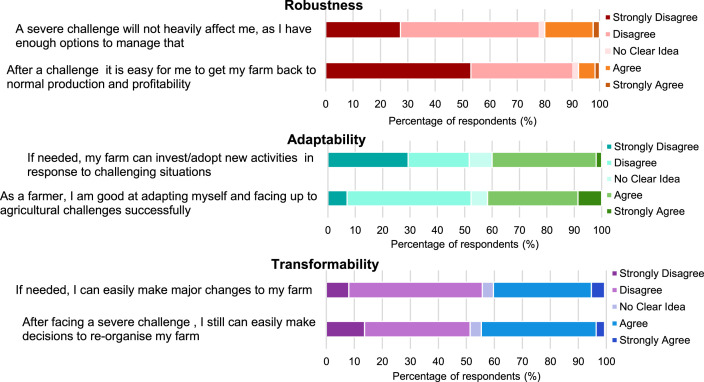
Farmers’ response to perceived resilience capacities.

There are three resilience capacities for adapting to diverse challenges: robustness, adaptability, and transformability [1,2]. Robustness refers to the system’s ability to withstand and recover from shocks; adaptability to its capacity to adjust while maintaining core functions; and transformability to its ability to undergo fundamental change when existing practices become unsustainable. Farmers' perceived resilience capacities were assessed using six statements (two statements for each capacity), guided by insights from previous studies [2,3], and a 5-point Likert scale (strongly agree to strongly disagree) was used to measure farmers' responses as illustrated in Fig. 3.

### 3.5 Resilience perceptions of farmers

**Fig. 4:**
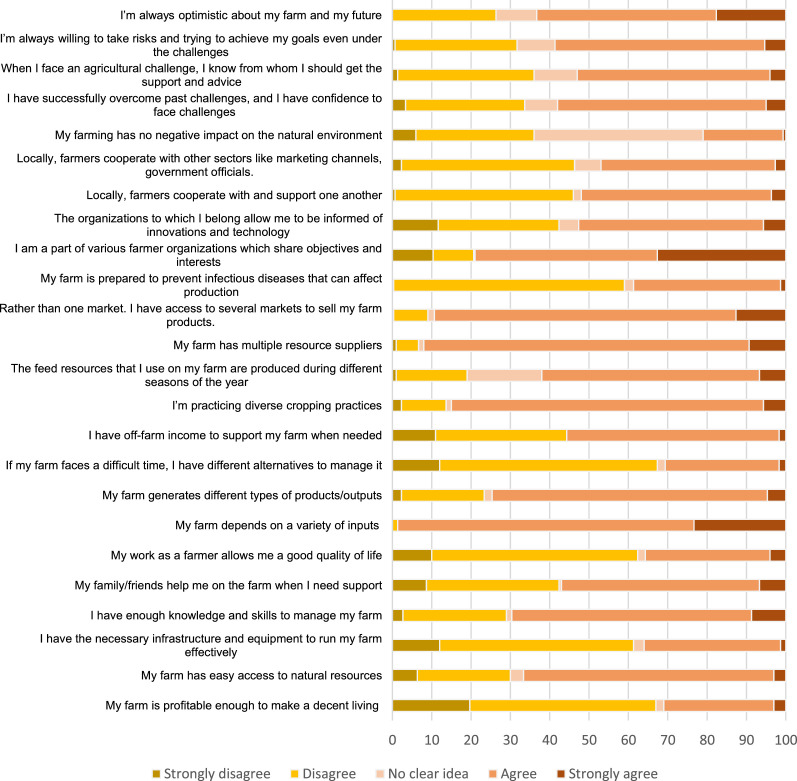
Farmers’ perceptions on resilience.

Farmers' resilience perceptions were assessed using 24 statements developed based on insights from previous studies [2,3]. The statements covered a range of resilience-related dimensions, including farm resources, diversity, management flexibility, social support, and psychological factors. Farmers indicated their level of agreement with each statement using a five-point Likert scale ranging from 1 (strongly disagree) to 5 (strongly agree). The distribution of responses is presented in Fig. 4.

### 3.6 Risk management strategies followed by farmers

**Fig. 5:**
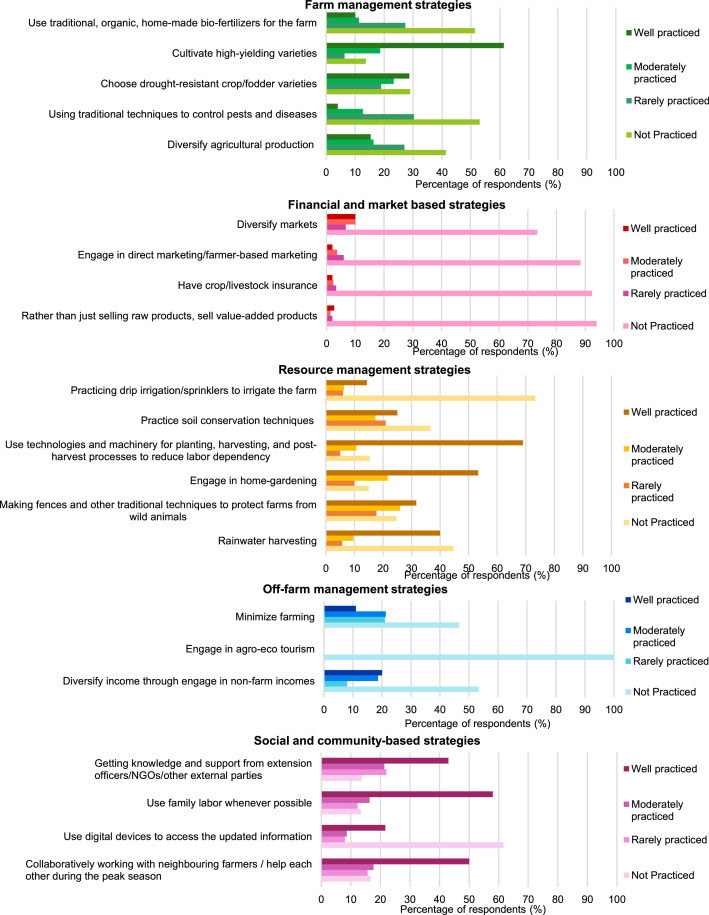
Farmers’ response on risk management strategies.

Twenty-two (22) risk management strategies were identified and grouped into five categories according to the type of management approach. During the survey, farmers were asked to indicate which risk management strategies they practice. Their responses were measured using a four-point Likert scale (0: Not practice, 1: Rarely practice, 2: Moderately practice, 3: Well practice) and illustrated in Fig. 5.

## 4 Experimental Design, Materials and Methods

Data collection was conducted in the Galgamuwa Divisional Secretariat, located in the Kurunegala District of the North-Western Province of Sri Lanka (7°59′11.4″N, 80°17′16.4″E). The area lies within the dry zone of Sri Lanka, characterised by a mean annual rainfall of <1750 mm. During the period 1989–2021, the mean annual precipitation in Galgamuwa was approximately 1380 mm [4]. Approximately 60–70% of the annual rainfall is received during the north-eastern monsoon (September–January), while pronounced dry periods occur from February to May and August to October. The average annual temperature was reported as 28.5 °C in 2024 [5]. The total land area of the Galgamuwa Divisional Secretariat is 272.19 km², with a population of approximately 67,660, of which 13,329 individuals are engaged in farming activities [6].

The area lies within the Mee Oya river basin, which is recognised as one of the most climate-vulnerable regions in the country. The region is characterised by a high density of small, seasonal village reservoirs and is increasingly affected by variable and unpredictable climatic conditions. Small-scale mixed farming systems are the dominant form of agriculture in the region and face multiple agricultural and socio-ecological challenges similar to those experienced by smallholder systems in other developing regions. Farmers typically cultivate a combination of paddy and seasonal field crops (maize, mung beans, chilli, low-country vegetables), and fruit crops, while also engaging in livestock rearing. These mixed systems are sensitive to environmental variability and institutional support structures, making them a valuable case study for examining resilience dynamics.

**Fig. 6:**
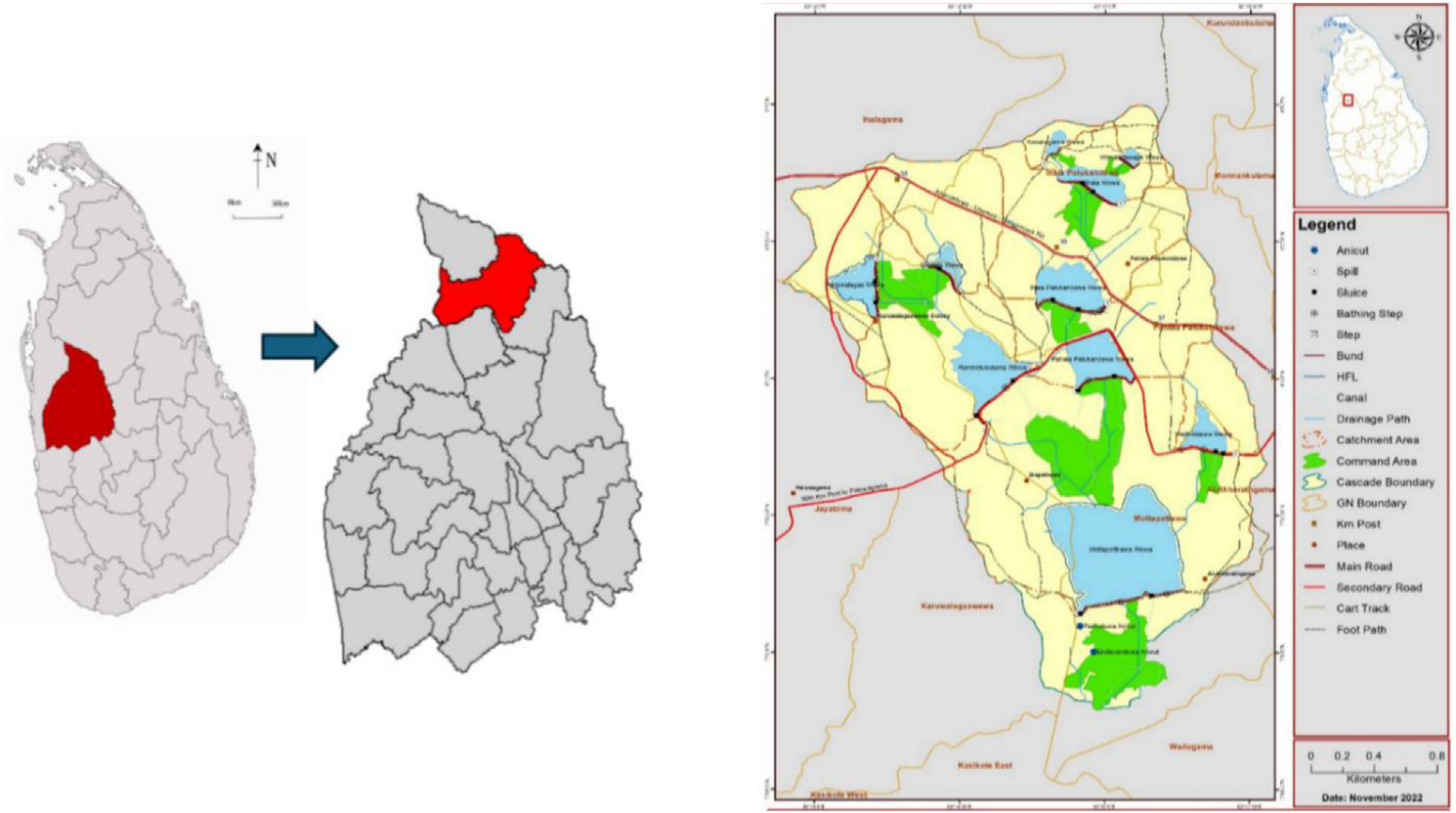
Study area; Kurunegala District and Galgamuwa divisional secretariat and Mottapeththewa cascade system in Galgamuwa divisional secretariat of Sri Lanka. (Source: climate resilient integrated water management project (CRIWMP), Ministry of Irrigation, Sri Lanka) [9].

Data collection was conducted in smallholder mixed farming systems within the Mottapeththewa cascade system in the Galgamuwa Divisional Secretariat (Fig. 6). Cascade systems are characterised by clusters of small, interconnected village reservoirs located within micro-catchments and hydrologically linked in a sequential network to store, regulate, and utilise water for agricultural production [7,8]. The cascade command area spans five village administrative divisions and twelve villages, covering an extent of approximately 1318.3 ha, and supports approximately 1300 farming households.

Data were collected through face-to-face surveys with 300 smallholder mixed farmers from twelve villages across five village administrative divisions within the *Galgamuwa* Divisional Secretariat using a pre-tested structured modified questionnaire from June 2025 to September 2025. The questionnaire was modified based on prior research conducted for the European farming systems [2,10]. The questions for each section were modified and developed as suitable for the rural, small-scale mixed farming systems based on the researchers’ expertise. The questionnaire included both closed-ended and open-ended questions. Closed-ended questions were primarily designed to measure respondents’ socio-demographic details, perceptions of agricultural challenges they face, risk management strategies they follow, and their perceived resilience capacities. To measure perceptions of agricultural challenges, they face five-point Likert scale was used (1: Not a challenge, 2: Light challenge, 3: Moderate challenge, 4: Considerable challenge, 5: Significant challenge). Risk management strategies were measured using a four-point Likert scale (0: Not practice, 1: Rarely practice, 2: Moderately practice, 3: Well practice), and farmers’ perceived resilience capacities were also measured by 5 point Likert scale (5: Strongly agree, 4: Agree, 3: No clear idea, 2: Disagree, 1:Strongly disagree). Surveys were conducted in the local language (Sinhalese). Ethics approval for data collection was obtained from the Deakin University Human Research Ethics Committee. Face-to-face surveys with farmers were conducted by three trained research assistants together with the researchers.

**Fig. 7:**
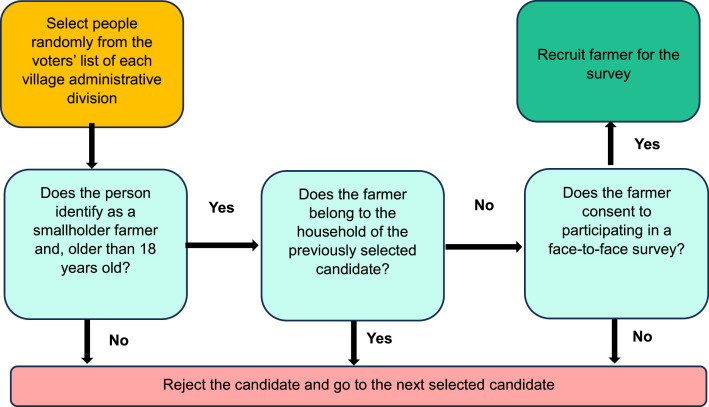
Participant selection process for face-to-face interview.

Before data collection, consent was obtained from the Galgamuwa Divisional Secretariat office. The total sample of 300 respondents was allocated proportionally across the 12 villages based on the number of farming households in each village, using information provided by the Galgamuwa Divisional Secretariat office. Within each village, farmers were randomly selected from the official voters' lists. If a selected individual was ineligible, declined to participate, or belonged to the same household as a previously selected respondent, another farmer was randomly selected from the same village until the predetermined sample size for that village was achieved. Eligibility criteria included being a small-holder farmer, at least 18 years of age, and willing to participate in the face-to-face survey. Only farmers were interviewed (not any other household members of the farm family), as the study aimed to capture farmers' lived experiences, perceptions, knowledge, and challenges. Before data collection, potential respondents were provided with a Plain Language Statement (PLS) explaining the research objectives and procedures, and their written consent was obtained (Fig. 7). The survey required approximately one hour to complete. The questionnaire was completed by the researchers and trained research assistants during the face-to-face interviews. Participants were provided with a monetary incentive in recognition of their time and contribution. All survey data were entered anonymously and analysed using Microsoft Excel. All codes for the survey questions are included in the first sheet of the data file (Microsoft Excel) submitted to the repository.

## Limitations

This dataset is based on cross-sectional, self-reported data from 300 smallholder farmers in the Galgamuwa area of Sri Lanka. Responses may be influenced by recent experiences and subject to recall and social desirability biases. The use of face-to-face surveys in a low-literacy context may introduce interviewer bias, despite efforts to standardise data collection through well-trained data enumerators. Minor inconsistencies may also arise from translation and interpretation, as the survey was conducted in the local language.

## Ethics Statement

Ethical approval for the study was obtained from the Deakin University Human Research Ethics Committee (Reference No 2025/HE000334). All research procedures followed approved ethical standards for studies involving human participants. Before conducting surveys, participants were informed of the research's purpose, the voluntary nature of their participation, and their right to withdraw at any time. Written informed consent was obtained from all participants before data collection commenced. To ensure confidentiality, all respondent identities were fully anonymised in the dataset, and no personally identifiable information was retained.

## Credit Author Statement

**Arani K. Ranasinghe:** Conceptualisation, Methodology, Data curation, Visualisation, Writing –original draft; **Lai Ming Lam:** Conceptualisation, Methodology, Writing –review & editing; **Kelly K. Miller:** Conceptualisation, Methodology, Writing –review & editing; **Brett A. Bryan:** Conceptualisation, Methodology, Writing –review & editing.

## Data Availability

Mendeley DataData on Challenges and Resilience Capacities of Smallholder Farmers in Sri Lanka (Original data). Mendeley DataData on Challenges and Resilience Capacities of Smallholder Farmers in Sri Lanka (Original data).
